# Ceramide-Induced Lysosomal Biogenesis and Exocytosis in Early-Onset Preeclampsia Promotes Exosomal Release of SMPD1 Causing Endothelial Dysfunction

**DOI:** 10.3389/fcell.2021.652651

**Published:** 2021-05-04

**Authors:** Leonardo Ermini, Abby Farrell, Sruthi Alahari, Jonathan Ausman, Chanho Park, Julien Sallais, Megan Melland-Smith, Tyler Porter, Michael Edson, Ori Nevo, Michael Litvack, Martin Post, Isabella Caniggia

**Affiliations:** ^1^Lunenfeld-Tanenbaum Research Institute, Sinai Health System, Toronto, ON, Canada; ^2^Institute of Medical Sciences, University of Toronto, Toronto, ON, Canada; ^3^Department of Physiology, University of Toronto, Toronto, ON, Canada; ^4^Sunnybrook Health Sciences Centre, Toronto, ON, Canada; ^5^Translational Medicine Program, Peter Gilgan Center, The Hospital for Sick Children, Toronto, ON, Canada; ^6^Department of Obstetrics and Gynecology, University of Toronto, Toronto, ON, Canada

**Keywords:** preeclampsia, lysosomes, exosomes, SMPD1, placenta

## Abstract

Aberrant ceramide build-up in preeclampsia, a serious disorder of pregnancy, causes exuberant autophagy-mediated trophoblast cell death. The significance of ceramide accumulation for lysosomal biogenesis in preeclampsia is unknown. Here we report that lysosome formation is markedly increased in trophoblast cells of early-onset preeclamptic placentae, in particular in syncytiotrophoblasts. This is accompanied by augmented levels of transcription factor EB (TFEB). *In vitro* and *in vivo* experiments demonstrate that ceramide increases TFEB expression and nuclear translocation and induces lysosomal formation and exocytosis. Further, we show that TFEB directly regulates the expression of lysosomal sphingomyelin phosphodiesterase (L-SMPD1) that degrades sphingomyelin to ceramide. In early-onset preeclampsia, ceramide-induced lysosomal exocytosis carries L-SMPD1 to the apical membrane of the syncytial epithelium, resulting in ceramide accumulation in lipid rafts and release of active L-SMPD1 *via* ceramide-enriched exosomes into the maternal circulation. The SMPD1-containing exosomes promote endothelial activation and impair endothelial tubule formation *in vitro*. Both exosome-induced processes are attenuated by SMPD1 inhibitors. These findings suggest that ceramide-induced lysosomal biogenesis and exocytosis in preeclamptic placentae contributes to maternal endothelial dysfunction, characteristic of this pathology.

## Introduction

Lysosomes are acidic organelles that degrade and recycle unwanted material and damaged intracellular components, including membranes, lipids and proteins (Mizushima et al., 2008; Luzio et al., 2009). Lysosomes are also subjected to lysosomal exocytosis (Medina et al., 2011). In the latter process, lysosomes are targeted to the plasma membrane where they fuse and release their contents outside the cells (Chieregatti and Meldolesi, 2005), thereby contributing to plasma membrane repair, cell signaling and immune responses (Rodriguez et al., 1997; Andrews, 2000; Bossi and Griffiths, 2005).

A gene network termed coordinated lysosomal expression and regulation (CLEAR) regulates lysosomal biogenesis and function (Sardiello et al., 2009). The master regulator of this network, Transcription Factor EB (TFEB), belongs to a class of transcription factors comprising the MiTF/TFE family (Palmieri et al., 2011). TFEB subcellular localization and function are tightly controlled by its phosphorylation status at specific serine residues, including S142 and S211 that are targets of rapamycin (mTOR). Phosphorylated TFEB is bound to 14-3-3 proteins that retain TFEB in the cytoplasm. Under stress conditions, cytosolic TFEB is dephosphorylated *via* inhibition of mTOR and concomitant activation of calcineurin phosphatase (Medina et al., 2015). Dephosphorylated TFEB translocates to the nucleus, where it binds to the palindromic motif (TCACGTGA) of CLEAR elements thereby inducing the transcription of an array of genes involved in lysosomal biogenesis and autophagy (Settembre et al., 2013; Martina and Puertollano, 2017). Disruption of TFEB function characterizes several neurodegenerative storage disorders and pharmacological induction of endogenous TFEB has been reported to be beneficial in correcting the disease’s phenotype (Dehay and Bezard, 2011; Medina et al., 2011; Spampanato et al., 2013; Polito et al., 2014; Xiao et al., 2014; Chauhan et al., 2015; Kilpatrick et al., 2016). TFEB’s action is not only limited to homeostasis and clearance of cells. Osteoclasts undergo a profound reorganization of their endo-lysosomal system during skeletal formation and remodeling that is mediated by TFEB (Ferron et al., 2013). Other studies have highlighted the contribution of TFEB in the transcriptional regulation of immune responses in macrophages (Pastore et al., 2016) and T lymphocytes (Huan et al., 2006; Samie and Cresswell, 2015). Moreover, dysregulation of members of the MiTF/TFE family can lead to different types of cancers (Slade and Pulinilkunnil, 2017). Thus, TFEB’s modulation of transcriptional networks is cell-type and context dependent.

Sphingolipids are an important class of bioactive lipids that include sphingosine, sphingosine-1-phosphate, ceramide, and sphingomyelin. We recently reported that preeclampsia (PE), a serious hypertensive disorder that complicates 5–8% of all pregnancies, can be regarded as a sphingolipid storage disorder (Melland-Smith et al., 2015). In PE, reduced acid ceramidase content and activity in conjunction with elevated ceramide *de novo* synthesis result in a build-up of ceramides in lysosomes of trophoblast cells, leading to increased autophagy, mitochondrial fission rates and necroptosis (Melland-Smith et al., 2015; Bailey et al., 2017; Ausman et al., 2018). Ceramides are important signal effector molecules in the cellular response to stress (Hannun, 1996); however, to date, their importance for lysosomal biogenesis during human placental development and disease remains to be established.

Herein, we examined the role of ceramide in lysosome formation and function in preeclamptic placentae. We show that ceramide is a powerful inducer of TFEB and consequently lysosomal biogenesis and exocytosis in trophoblast cells. Furthermore, we demonstrate that the increase of lysosomes in trophoblast cells from PE placentae is accompanied by augmented lysosomal exocytosis in the syncytium, the trophoblast layer where ceramide accumulates. Exuberant lysosomal exocytosis in PE causes lysosomal sphingomyelin phosphodiesterase 1 (L-SMPD1) to accumulate in syncytial plasma membrane lipid rafts. Subsequent release of active L-SMPD1 *via* exosomes by the syncytial cells into the maternal circulation contributes to the ceramide-induced endothelial dysfunction seen in PE women.

## Materials and Methods

### Placental Tissue Collection

The study was approved by the Mount Sinai Hospital Research Ethics Board (REB number: 11-0287-E). Informed consent was obtained from all subjects, and placentae and maternal plasma were collected by the Research Centre for Women’s and Infants’ Health (RCWIH) Biobank. Severe early-onset PE subjects (*n* = 54) were selected based upon the American College of Obstetrics and Gynecology (ACOG) criteria (American College of Obstetricians et al., 2013). Typically, in the early-onset preeclamptic (E-PE) cases clinical manifestations occurred within 2 weeks prior to delivery. Only singleton pregnancies were included in the study. Pregnancies affected by fetal malformations, chromosomal abnormalities, chorioamnionitis and from smokers and substance abusers were excluded. Placental samples from normotensive gestational age-matched pre-term control (PTC, *n* = 48) and term control (TC, *n* = 14) deliveries from healthy pregnancies that did not exhibit clinical symptoms of PE or other pregnancy-associated disorders, were included as controls. Cervical incompetence, idiopathic labor, and preterm premature rupture of membranes were causative for preterm deliveries. Clinical parameters of subjects are listed in Table 1.

**TABLE 1:** Clinical parameters of the study population.

	E-PE (54)	PTC (48)	TC (14)
**Mean GA at delivery** (weeks)	29.28 ± 0.40	30.58 ± 0.95	38.83 ± 0.36
**Blood Pressure**			
*Systolic*	166.22 ± 4.87	113.00 ± 4.47	113.50 ± 9.19
*Diastolic*	101.53 ± 3.1	73.25 ± 3.20	71.00 ± 1.41
**Proteinuria**	3.80 ± 0.10	Absent	Absent
**Fetal Weight** (g)	1084.22 ± 71.80	1493.33 ± 54.73	3272.14 ± 171.15
**Fetal Sex** (%)	M: 75^∗^	M: 50	M: 28.5
	F: 25^†^	F: 50	F: 71.5
**Mode of Delivery** (%)	CS 93.00^‡^	CS 40	CS 71.5
	VD 7.00^§^	VD 60	VD 28.5

Data are presented as mean ± standard deviation. *M: Male. ^†^F: Female. ^‡^CS: Caesarian section. ^§^VD: Vaginal delivery. PE, preeclampsia; PTC, preterm control; TC, term control.

### Ceranib-2 Mice

Animal studies were approved by the Animal Care Committee of the Hospital for Sick Children (Toronto, Canada). CD1 mice were obtained from Charles River (St. Constant, QC, Canada). During pregnancy, mice were injected intraperitoneally daily (from E7.5 till E13.5) with Ceranib-2 suspended in dimethyl sulfoxide (DMSO) (20 mg/kg; Cayman Chemical, 11092) as previously reported (Melland-Smith et al., 2015). Mice injected with DMSO alone were used as controls. Placentae were obtained at E13.5 and snap frozen for WB analysis or processed for histochemical analyses.

### Cell Lines and Primary Cultures

#### Primary Cytotrophoblast Cells Isolation

Term placentae (*n* = 3) obtained from uncomplicated pregnancies undergoing elective cesarean sections were processed for primary trophoblast cells isolation as previously described (Bailey et al., 2017; Ausman et al., 2018). In brief, placental tissue was cut into small pieces and digested in Dulbecco’s modified Eagle’s medium (DMEM, GIBCO-BRL, 11039-021) containing 0.05 mM trypsin (GIBCO 27250-018; Invitrogen) and 0.008 mM DNase I (SIGMA DN25; Sigma-Aldrich Corp. St. Louis, MO, United States) at 37°C. The cell suspension was then filtered through a 70 μM nylon sieve (Becton, Dickson and Company, Franklin Lakes, NJ, United States) and subjected to centrifugation on a discontinuous 5–70% Percoll gradient (GE Healthcare, Little Chalfont, United Kingdom). The layer corresponding to 35–45% of Percoll was collected and washed with DMEM. Isolated cells were counted and plated at a density of 1 × 10^7^ cells in six-well plates in DMEM media containing 10% (v/v) fetal bovine serum (FBS) and 1% (v/v) penicillin–streptomycin (Wisent Inc., St Bruno, Canada). Primary isolated trophoblast cells were maintained at their normoxic conditions of 8% O_2_/% CO_2__/_, 87% N_2_.

#### Human Microvascular Endothelial Cells

Human Microvascular Endothelial Cells (HMVEC), immortalized with human telomerase catalytic protein (Shao and Guo, 2004), were a gift from Dr. ON (Sunnybrook Health Sciences Centre, Toronto, ON, Canada). HMVECs from passage 20–40 were cultured in 6-well plates in Endothelial Cell Growth Base Media containing Endothelial Cell Growth Supplement (RD systems, Oakville, Canada) in standard (ambient air and 37°C) culture conditions.

#### Choriocarcinoma JEG3 Cells

Choriocarcinoma JEG3 cells (ATCC^®^ HTB-36^TM^, ATCC), authenticated by short tandem repeat genotyping, were cultured in ambient air at 37°C in Eagle’s Minimum Essential Medium (EMEM) (ATCC, 30-2003) containing 10% (v/v) FBS and 1% (v/v) penicillin–streptomycin (Wisent Inc., St Bruno, Canada). Upon reaching 80% confluency in six-well plates, cells were washed and then cultured in EMEM for 6 h with either 20 μM CER 16:0 (Enzo Life Sciences, BML-SL115), a treatment that triggers autophagy in JEG3 cells (Melland-Smith et al., 2015), 25 μM 2-oleoylethanolamine (2-OE; Invitrogen, 0383), an acid ceramidase inhibitor that increases ceramide content in these cells thereby inducing autophagy (Melland-Smith et al., 2015) or EtOH and DMSO, respectively, as vehicles. JEG3 cells were treated for 24 h with and without 2.5 mM sodium nitroprusside (SNP), a nitric oxide donor known to activate the Fenton reaction thereby inducing oxidative stress via reactive oxygen species production (Myatt and Cui, 2004). Concentrations for CER 16:0, 2-OE, and SNP were based on our previous published studies (Melland-Smith et al., 2015; Ausman et al., 2018). Cell were either collected for Western blot (WB) analysis or fixed with 4% (v/v) paraformaldehyde for immunofluorescence (IF) analysis.

For Fluorescein isothiocyanate–dextran incorporation, JEG3 cells cultured on coverslips in six-well plates were incubated with 2.5 mg/ml of fluorescein isothiocyanate (FITC)–dextran (Millipore Sigma^®^, 46945, Toronto, Canada) at standard conditions. After 6 h of incubation (optimal time for FITC-dextran loading), cells were washed and cultured in EMEM for 6 h with 20 μM CER 16:0 or vehicle EtOH. Cells were fixed with 4% (v/v) paraformaldehyde for IF while conditioned media was collected for luminometric analysis (Tecan Life Sciences).

For silencing experiments, JEG3 cells (60–80% confluency) were transfected with either 60 nM of Silencer^®^ select siRNA targeted against *TFEB* (ThermoFisher Scientific^®^, Ottawa, Canada), or scrambled control siRNA sequences using a jetPRIME^®^ transfection protocol (Polyplus Transfection^®^, 89129-922, New York, United States). Cells were cultured in EMEM at ambient air and 37°C and collected 24 h later for WB analysis.

Measurement of the intracellular calcium variation with Fura-2 (ThermoFisher Scientific^®^, Ottawa, Canada; Catalog: F1201) on JEG3 cells treated with CER 16:0 or ethanol was performed as described by Martinez (Martinez et al., 2016).

### Gene Reporter Assay

Choriocarcinoma JEG3 cells were plated at a density of 2 × 10^5^ cells/well, cultured for 24 h at 37°C in ambient air and then transiently transfected with SMPD1-Luciferase, in combination with empty vector (EV) pcDNA3.1 or pcDNA-TFEB wild type (WT). Renilla luciferase was used as a non-TFEB-responsive plasmid for normalizing transfection efficiencies and monitoring cell viability. The total amount of plasmid DNA was normalized to 1.0 μg/well using empty pcDNA3.1 vector. After 24 h of treatment, cells were harvested and processed according to the manufacturer protocol LightSwitch Luciferase^®^ Assay System kit (SWITCHGEAR Genomics, Carlsbad, CA, United States). Luciferase activity was measured using a luminometer (Tecan Life Sciences, Männedorf, Switzerland). SMPD1-Luciferase activity was normalized to constitutive RenSp-driven promoter Renilla-luciferase. Experiments were repeated three times in triplicate.

### Chromatin Immunoprecipitation Assay

Chromatin immunoprecipitation (ChIP) of TFEB was performed in E-PE (*N* = 4) and PTC (*N* = 4) placentae and in JEG3 cells exposed to 20 μM CER 16:0 or control vehicle, using the EpiQuik Tissue ChIP Kit (EpiGentek, Farmingdale, NY, United States) according to the manufacturer’s instructions. Briefly, ∼50 mg of placental tissue was homogenized using a Dounce homogenizer and cross-linked. JEG3 cells were lysed and DNA was sheared by sonication prior to cross-linking. Samples were then incubated with 2 μg anti-goat ChIP-grade TFEB antibody per 25 μg chromatin to immunoprecipitate the protein-DNA complexes. Non-immune mouse IgG was used as a negative control, while RNA Polymerase II enrichment at the *GAPDH* promoter was used as positive control. Protein-DNA complexes were purified, and DNA was extracted. The purified DNA was quantified by qPCR using the Sybr Green^®^ qPCR mastermix (Applied Biosystems (ABI), Foster City, CA, United States), employing primers targeting two distinct sites along the SMPD1 promoter (Table 2 depicts primer sequences).

**TABLE 2:** Oligonucleotide sequence for primers used in ChIP-qPCR analysis for TFEB binding to *SMPD1* promoter.

SMPD1 promoter region between −300 and −200 bp	SMPD1 promoter region between −1,000 and −900 bp
5′-ctggtgacctcagggagagt-3′	5′-taacggctttgggagagagg-3′
5′-ccttcctcttcctctgatctc-3′	5′-ggggctcagaaatccatacc-3′

### Exosome Isolation and Uptake

Exosome isolation and characterization from maternal plasma and from conditioned media of JEG3 cells treated with either CER 16:0 or EtOH were performed as reported previously (Ermini et al., 2017). See detailed procedures in Supplemental Methods. Total and placental exosomes were subjected to lipid and WB analyses. Placental exosomes were then isolated by selective immunoprecipitation using anti-human PLAP. Exosome purity was verified by particle size (NanoSight particle analyzer; Malvern Instruments Ltd., Malvern, United Kingdom), and immunoblotting for CD63 (generic marker for exosomes) and placental alkaline phosphatase (PLAP, marker for placental-derived exosomes) (Ermini et al., 2017; Salomon et al., 2017).

Sphingomyelin phosphodiesterase 1 activity in exosomes isolated from conditioned media of JEG3 cells treated with 20 μM CER 16:0 (ExoCer) or EtOH (ExoV) for 6 h was measured using an Echelon SMPD1 assay kit according to the manufacturer’s protocol (Echelon Bioscience, K-3200) as previously reported (Melland-Smith et al., 2015).

To examine exosome uptake by HMVECs, exosomes were labeled in the dark for 5 h with PKH67, a green fluorescent dye with a long aliphatic tail that incorporates into the exosome lipid membrane, using the PKH67 Fluorescent Cell linker Kit (Sigma, PKH67GL-1KT). The staining reaction was ended by adding an equal volume of 1% (w/v) bovine serum albumin to bind excess dye. Labeled exosomes were subsequently pelleted (120,000*g* for 60 min) and resuspended in PBS. Spin and wash cycles were performed at least three times. At 60–70% confluency, HMVECs were incubated with the labeled exosomes for 30 min, 1, 3, and 6 h, respectively. After the optimal uptake time for exosomes was established using a Leica SD6000 spinning disk confocal microscope (Leica Camera, Wetzlar, Germany), HMVECs were exposed for 3 h to 2.0 × 10^6^ ExoCer and ExoV exosomes isolated from conditioned media of JEG3 cells treated for 6 h with 20 μM CER 16:0 or vehicle EtOH, respectively. Cells were then collected for WB analysis, or fixed with 4% (v/v) paraformaldehyde for IF analysis.

### Tubule Formation Assay

For the tubule formation assay, 24-well plates were coated with 200 μl (concentration 10 mg/ml) of Matrigel (Corning^®^ Matrigel^®^ Matrix, 354234). The matrix was allowed to solidify in the incubator for 30 min following which 1 × 10^5^ HMVECs/well were seeded. Cells were then exposed to either 20 μM CER 16:0, 25 μM2-OE or respective vehicle EtOH or DMSO. HMVEC were also exposed to 2.0 × 10^6^ ExoCer and ExoV exosomes that were isolated from conditioned media of JEG3 cells treated for 6 h with 20 μM CER 16:0 or vehicle EtOH, respectively. The exosome exposure occurred in presence and absence of 25 μM of Imipramine (Sigma-Aldrich, St. Louis, United States, #) or 10 μM Fluoxetine (Sigma-Aldrich, St. Louis, United States, #F132), inhibitors of SMPD1 activity (Justice et al., 2018). Images of tubule structures were acquired and the Angiogenesis Analyzer plugin of ImageJ was used for quantification of number of branches and the total length of the tubular network (DeCicco-Skinner et al., 2014).

### Transmission Electron Microscopy

PE (*n* = 5) and PTC (*n* = 4) placental tissues and primary isolated cytotrophoblasts exposed for 6 h to 20 μM CER 16:0 or vehicle EtOH at 8% O_2_ were processed for transmission electron microscopy (TEM) analysis as previously described (Ausman et al., 2018). Placental tissue and primary isolated cytotrophoblast cells fixed in 2% (v/v) glutaraldehyde in 0.1 M cacodylate buffer (pH 7.3) were processed by the Nanoscale Biomedical Imaging Facility, The Hospital for Sick Children, Toronto. Imaging was conducted on a FEI Technai 20 Transmission Electron Microscope. Primary and secondary lysosomes were identified and counted by two individuals, blinded to the study, independently.

### Immunofluorescence Analysis

Immunofluorescence staining and quantification were performed as previously described (Bailey et al., 2017). Lysosomal activity was monitor using LysoTracker^®^ Red (Invitrogen^®^, L7528) as previously reported (Melland-Smith et al., 2015). For placental tissue sections, 10 mM sodium citrate, pH 6.0 was used for antigen retrieval, followed by treatment with Sudan Black (Sigma, 199664; 0.3% Sudan Black in 70% ethanol) to quench endogenous fluorescence. Following experimental treatments, 4% (v/v) paraformaldehyde (Sigma^®^, F8775) was used to fix cells for 15 min at 37 °C. Cells and tissue were permeabilized with 0.2% (v/v) Triton X-100 for 5 min, rinsed with phosphate-buffered saline (PBS) and treated with 5% (w/v) normal horse serum (NHS) (Sigma^®^, H0146) diluted in PBS for 1 h at room temperature to block non-specific binding. Primary antibodies were diluted in antibody diluent (0.4% sodium azide, 0.625% gelatin) and 5% (w/v) NHS, and added to samples for incubation overnight at 4°C. For negative controls, the primary antibody was replaced with non-immune rabbit IgG (Santa Cruz Biotechnology^®^, sc-2027). FITC-conjugated secondary antibodies, diluted in antibody diluent at a concentration of 1:200, were added for 1 h, followed by three additional PBS washes. Prior to mounting on glass slides with Immuno-Mount^TM^ (Thermo Fisher Scientific^®^), samples were treated with 4’,6-diamino-2-phenylindole (DAPI; Sigma, D9542) to detect nuclei. Lysosomal activity was monitor using LysoTracker^®^ Red (Invitrogen^®^, L7528) as previously reported (Melland-Smith et al., 2015). The lysotracker probe was dissolved in DMSO. After 2-OE or CER C16:0 treatments, cells were incubated with either 50 nM LysoTracker solution or equivalent volume of vehicle DMSO for 1 h at 37°C and fixed with 4% (v/v) formaldehyde. Cells were then washed in PBS and nuclei were subsequently counterstained with DAPI.

Immunofluorescence images were viewed and captured using a Quorum (Guelph, Ontario, Canada) WaveFX Spinning Disc Confocal System with optimized Yokogawa CSU X1, Hamamatsu EM-CCD digital camera Image EM (C9100-13), and Leica DMI6000B inverted research grade motorized microscope run by Volocity 6.3 Acquisition software (Improvision/PerkinElmer, Waltham, MA, United States). IF quantification was performed using Volocity Software to determine Mean Fluorescent Intensity.

### Flow Cytometry Analysis

To measure endothelial activation, HMVECs were harvested, resuspended in staining buffer (HBSS supplemented with 2% (v/v) FBS and 10 mM HEPES) and incubated for 30 min with antibodies validated for flow cytometry at a 1:100 dilution. Anti-human CD54-BV421 and CD146-PE were purchased from BD Biosciences. Stained cells were washed, resuspended in fresh staining buffer followed by flow cytometry analysis and analyzed using a Beckman Coulter Galios flow cytometer with data analysis completed using Kaluza software (Beckman Coulter, Mississauga, ON, Canada).

To quantify lysosomal volume changes, JEG3 cells were incubated with either vehicle EtOH, 20 μM of CER16:0, or 100 nM of Bafilomycin A (Millipore Sigma^®^, 46945, Toronto, Canada) for 6 h. Cells were then treated with 1 μM Lysotracker Red DND-99 (ThermoFisher Scientific^®^, Ottawa, Canada) for 45 min or 1 μg/ml acridine orange (ThermoFisher Scientific^®^, Ottawa, Canada) for 15 min, respectively. Cells were harvested, centrifuged at 300 *g* for 5 min and resuspended in staining buffer (PBS supplemented with 2% (v/v) FBS) with 1 μg/ml DAPI (viability dye control; Sigma, D9452) for 5 min. Stained cells were then washed, and resuspended in fresh staining buffer followed by flow cytometry analysis as described above. In all experiments, unstained cells were used as negative controls for proper gating and voltage setting as per manufacturer’s recommendations.

### Isolation of Lysosomes, Placental Apical Microvillous and Detergent-Resistant Membranes

Lysosomes, apical microvillous and detergent-resistant membranes (PE = 8, PTC = 7, TC = 7) were prepared from fresh human placentae as previously reported (Ermini et al., 2017). Villous tissue (0.5 g) was dissected into small pieces, washed with saline and homogenized in three volumes of ice-cold buffer A (250 mM sucrose, 0.7 × 10^–3^ mM pepstatin, 1.1 × 10^–3^ mM leupeptin, 0.8 × 10^–3^ mM antipain, 80 × 10^–6^ mM aprotinin and 10 mM Tris-HEPES, pH 7.4). The homogenate was spun at 5,860*g* for 15 min and the collected supernatant was centrifuged at 10,000 *g* for another 15 min. Lysosomes were then pelleted at 25,000*g* for 15 min. The remaining supernatant was centrifuged at 124,000 *g* for 30 min in a Beckman TL-100 ultracentrifuge and the membrane-enriched pellet was suspended in buffer A using a glass-teflon homogenizer. Magnesium chloride was added to separate the apical microvillous membranes from the basal membranes (Jimenez et al., 2004). The suspension was centrifuged at 2,500 *g* for 10 min and the apical microvillous membranes were pelleted at 12,100*g* for 70 min. The lysosomal and apical membrane pellets were resuspended in 300 mM sucrose, 20 mM Tris-maleate, pH 7.4.

### Lipid Mass Spectral Analysis

Soluble and insoluble apical membranes and exosomes from PE, PTC, and TC placentae were processed for lipid analysis (Melland-Smith et al., 2015; Ermini et al., 2017). Following lipid extraction, ceramides and cholesterol were quantified using high performance liquid chromatography and tandem mass spectrometry (LC-MS/MS) at the Analytical Facility for Bioactive Molecules of the Hospital for Sick Children, Toronto, ON, Canada.

### Immunohistochemistry and Western Blot Analysis

IHC staining and WB analyses in placental tissue and cell lines were performed as previously described (Melland-Smith et al., 2015; Bailey et al., 2017).

PE and PTC snap-frozen tissues were crushed in liquid nitrogen and homogenized in RIPA buffer (150 mM NaCl, 50 mM Tris, 1% NP-40, pH 7.5). Cultured cells were lysed in RIPA buffer and placed on ice for 1 h. The tissue homogenate and cell lysates were centrifuged and supernatant subjected to protein quantification prior to WB using the Bradford protein assay (BioRad^®^, 500-0006). Fifty μg of proteins were subjected to sodium dodecyl sulfate–polyacrylamide gel electrophoresis and then transferred onto methanol-hydrated polyvinylidene fluoride membranes. The membranes were pre-incubated in 5% (w/v) non-fat milk dissolved in tris-buffered saline (TBST) for 1 h and left overnight in primary antibody at 4°C. Secondary antibody conjugated to horseradish peroxidase (HRP) was added for 1 h at room temperature. Blots were imaged using chemiluminescence ECL-plus reagent (PerkinElmer Inc., NEL103001EA) and X-ray film (GE Healthcare). Densitometric analysis of WB was performed using ImageLab^®^ software (Bio-Rad, Hercules, CA, United States). Samples were normalized to either ACTB, GAPDH or Stain Free Blot (SFB).

### Antibodies

Anti-LAMP1 (rabbit, WB 1:3000, IF 1:500; ab2971) was acquired from Millipore Sigma^®^ (Toronto, Canada). Anti-TFEB antibody (rabbit, WB 1:2000, IF 1:500; ab174745) was purchased from Abcam^®^ (Toronto, Canada) and ChIP-grade TFEB antibody (goat, ChIP 1:10; NB-100-1030) was from Novus Biologicals^®^ (Littleton, United States). Antibodies against ICAM-1 (mouse, IF 1:200 WB: 1:500; sc-18853), ACTB (mouse, WB 1:2000; sc-47778), SMPD1 (rabbit, IF 1:200, WB 1:350; sc-11352), CD63 (mouse, WB 1:100; sc-5275) and CD34 (mouse, IHC 1:100) were obtained from Santa Cruz Biotechnology^®^ (Dallas, TX, United States). Anti-PLAP (rabbit, WB 1:2000, IP 1:40; ab198388) was acquired by Abcam^®^. Anti-ceramide IgM (IF 1:200, MAB-0013) was purchased from Glycobiotech^®^ (Seekoppel, Germany). Anti-CD31 (rabbit, IHC 1:50) was from Cell Signalling Technology (Beverley, MA, United States) and anti-TSG101 (rabbit, WB 1:500) from Thermo Fisher (Carlsbad, CA, United States). Secondary antibodies included goat anti-rabbit IgG-HRP (WB 1:3000; 111-035-144) and goat anti-mouse IgG-HRP (WB 1:2000, #1706516) and were purchased from Jackson ImmunoResearch Laboratories^®^ (West Grove, PA, United States) and BioRad^®^ (Hercules, CA, United States), respectively. For IF, Alexa Fluor^®^ 488 donkey anti-rabbit IgG (A21206), Alexa Fluor^®^ 594 donkey anti-mouse IgG (A21203), were purchased from ThermoFisher Scientific^®^ (ThermoFisher Scientific^®^, Ottawa, Canada).

### Statistical Analysis

All data are expressed as mean ± SEM. Statistical analysis was performed using GraphPad Prism 5 software. Comparison of data between two groups was done using two-tailed unpaired Student *t*-test. For comparison between multiple groups, Kruskal-Wallis test with a *post hoc* Dunn’s Multiple Comparison Test was performed. Significance was denoted as ^∗^*P* < 0.05, ^∗∗^*P* < 0.01, and ^∗∗∗^*P* < 0.001.

## Results

### Preeclamptic Placentae Exhibit Heightened Lysosome Biogenesis and TFEB Expression

**FIGURE 1:**
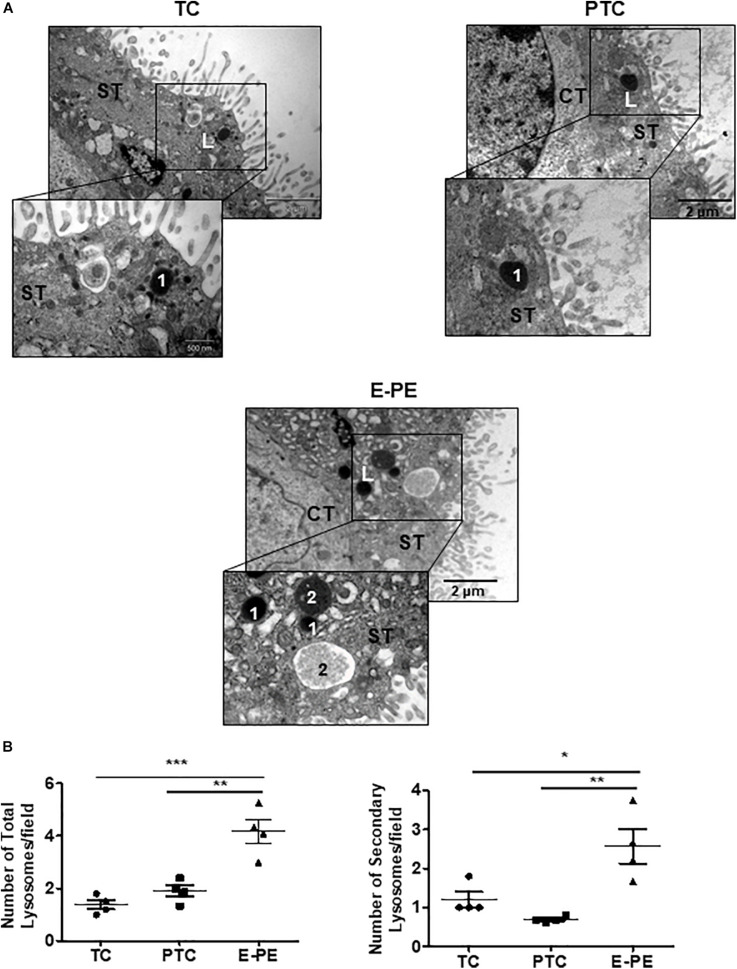
Lysosome morphology in E-PE and control placentae. **(A)** Representative TEM images of syncytiotrophoblast cells from TC, PTC and PE placental sections. L, lysosomes; 1 primary lysosome; 2 secondary lysosome. CT, cytotrophoblast; ST, syncytiotrophoblast. **(B)** Number of total and secondary lysosomes in syncytiotrophoblast cells from PCT and PE placentae (PE, *N* = 4; PTC, *N* = 4; **P < 0.01 compared to PTC).

**FIGURE 2:**
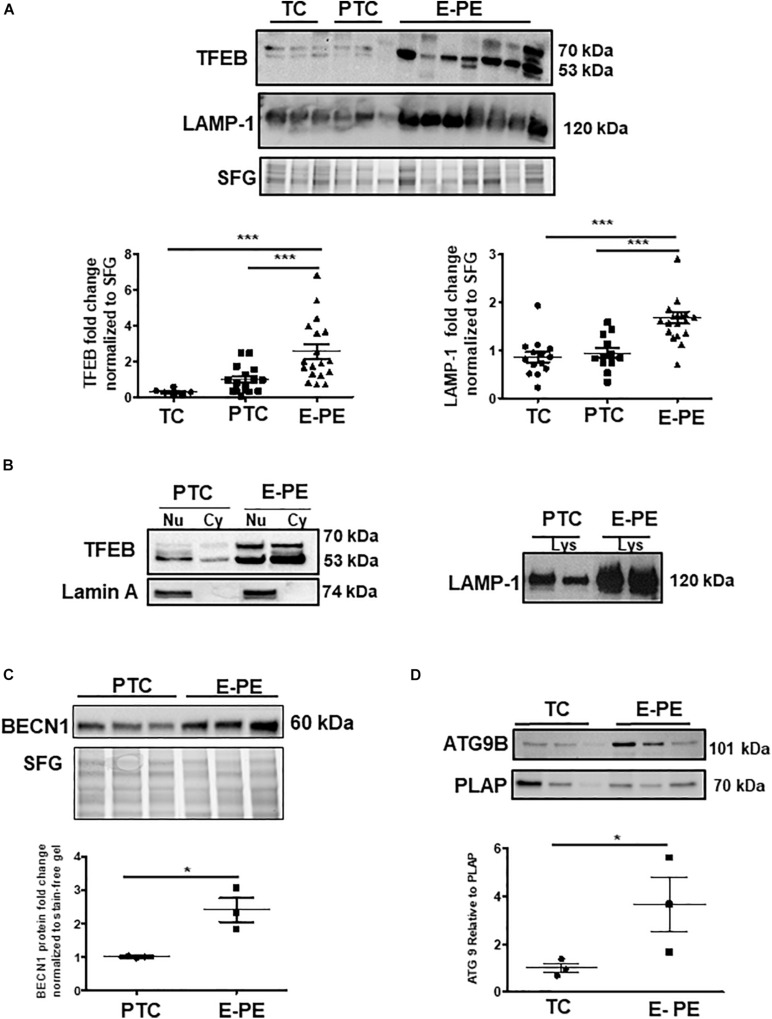
Lysosome biogenesis is increased in E-PE placentae. **(A)** Representative WB and corresponding densitometry of TFEB and LAMP-1 in E-PE, PTC, and TC placentae normalized to total protein in stain free gel (SFG) (TFEB: E-PE *N* = 18, PTC *N* = 16, TC *N* = 6, ***P < 0.001 E-PE compared to PTC and TC); LAMP-1: E-PE *N* = 19, PTC *N* = 12, TC *N* = 14, ***P < 0.001 E-PE compared to PTC and TC). (**B, left panel**) WB for TFEB and Lamin A in nuclear (Nu) and cytoplasm (Cy) enriched fractions; (**B, right panel**) WB for LAMP1 in lysosomal lysates from PTC and E-PE placentae. **(C)** WB of Beclin-1 (BECN1) in PTC and E-PE placentae (PE, *N* = 7; PTC, *N* = 7). **(D)** Representative WB for ATG9b and corresponding densitometry in lysates of syncytial cells isolated from term and PE placentae. PLAP was used as a syncytial marker and loading control. (PE, *N* = 3; TC, *N* = 3; *P < 0.05 compared to TC). Dotted line: lanes were run on the same gel but were non-contiguous. Data are expressed as mean ± SEM.

Ultrastructural analysis revealed a significant increase in the total number of lysosomes in the syncytiotrophoblast layer of E-PE compared to both PTC and TC placentae (Figures 1A,B). Lysosomes are typically distinguished as primary (electron-dense organelles) and secondary lysosomes (less electron-dense vacuoles). The increased number of total lysosomes in E-PE syncytiotrophoblasts relative to both PTC and TC was largely due to an increase in secondary lysosomes (Figures 1A,B). We also found a significant rise in secondary lysosomes in E-PE cytotrophoblasts (Supplementary Figure 1A), although markedly less than seen in E-PE syncytiotrophoblasts. Next, we examined the expression of TFEB, the master regulator of lysosomal biogenesis and function (Palmieri et al., 2011; Settembre et al., 2013). Western blotting (WB) showed a significant increase in TFEB (predicted MW is 53 kDa) in E-PE compared to PTC and TC placentae (Figure 2A). RNA silencing of *TFEB* in trophoblastic JEG3 cells and subsequent WB for TFEB verified the 53 kDa band as TFEB (Figure 5A). Immunoblotting for lysosomal-associated membrane protein 1 (LAMP-1), a specific lysosomal marker, demonstrated a significant increase in LAMP-1 levels in E-PE compared to PTC and TC placentae (Figure 2A), in line with the ultrastructural data. Immunofluorescence confocal (IF) analysis confirmed strong immune-positive staining for LAMP-1 in the syncytium (Supplementary Figure 1B). IF analysis showed that TFEB primarily localized to the syncytium where it was markedly increased in PE placentae (Supplementary Figure 1C). Furthermore, WB for TFEB of isolated nuclear, cytoplasmic and lysosomal fractions showed an increase of TFEB in E-PE placentae (Figure 2B). In addition to the lysosomal pathway, TFEB regulates the transcription of a number of autophagy-related genes (Palmieri et al., 2011; Settembre et al., 2011). Hence, we investigated levels of downstream TFEB target, Beclin-1 (BECN1) (Palmieri et al., 2011) and confirmed its upregulation in E-PE versus PTC placentae (Figure 2C). Since TFEB localized mainly to the syncytium (Supplementary Figure 1C), we isolated that layer and probed it for ATG9b, another well-known TFEB autophagy target (Palmieri et al., 2011; Settembre et al., 2011). Indeed, ATG9b levels were increased in the syncytial layer of E-PE compared to TC placentae (Figure 2D).

### Ceramide Promotes TFEB-Induced Lysosomal Biogenesis and SMPD1 Expression

**FIGURE 3:**
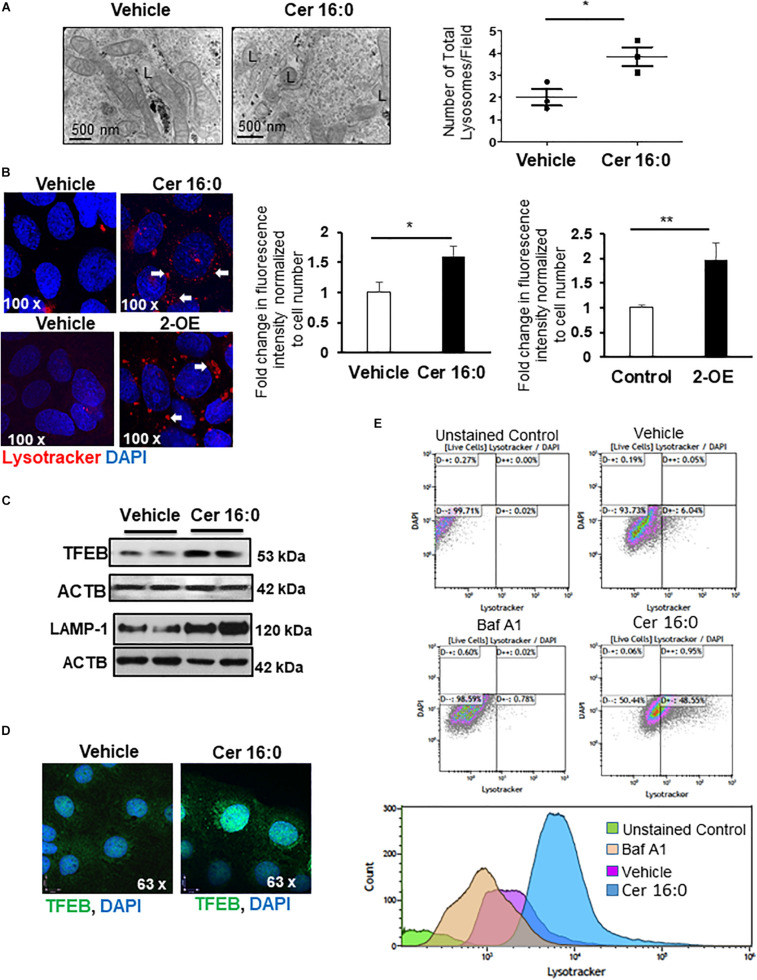
Ceramide induces lysosome biogenesis. **(A)** Representative TEM images and corresponding number of total lysosomes in primary isolated trophoblast cells treated with 20 μM CER 16:0 or EtOH vehicle (*N* = 3 experiments with freshly isolated cells; *P < 0.05). L: lysosomes. **(B)** LysoTracker^®^ Red IF of JEG3 cells cultured in the presence or absence of 20 μM CER 16:0 or 25 μM 2-OE and corresponding fold change in fluorescence intensity (*N* = 3 separate experiments; *P < 0.05, **P < 0.01 compared to vehicle control). Arrow: LysoTracker^®^ Red reactivity. **(C)** Representative WB for TFEB and LAMP1 in JEG3 cells treated with 20 μM CER 16:0 or vehicle. ACTB used as loading control. **(D)** IF analysis of TFEB (green) in JEG3 cells following exposure to 20 μM CER 16:0 or EtOH vehicle. Nuclei were visualized with DAPI (blue). Data are expressed as mean ± SEM. **(E)** Representative flow cytometry density and volume plots of Lysotracker^®^ Red stained JEG3 cells exposed for 6 h to either EtOH vehicle, 100 nM Bafilomycin (Baf A_1_) or 20 mM CER 16:0. Unstained cells were used for gating. Note that DAPI staining, used to mark viability of live cells, indicate no significant shifts between either unstained cells or cells exposed to vehicle EtOH, CER16:0 or Baf A_1_.

**FIGURE 4:**
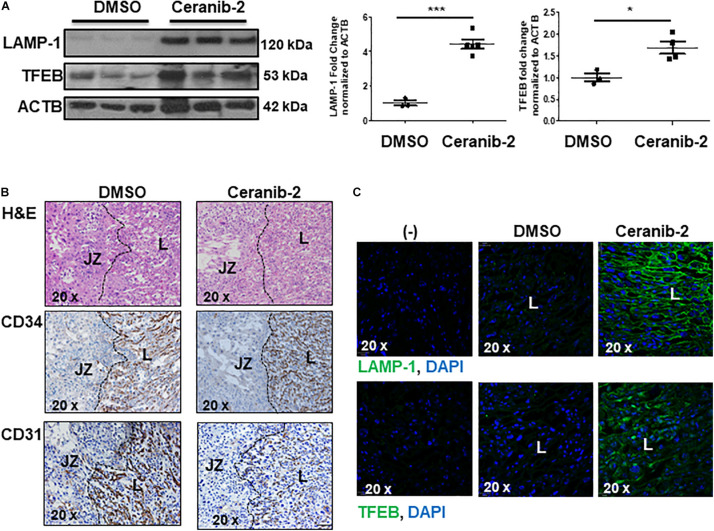
Inhibition of acid ceramidase with Ceranib-2 in pregnant mice increases placental lysosomal biogenesis **(A)** Representative WB for LAMP-1 and TFEB and corresponding densitometry in placental lysates from CD1 mice injected with ceranib-2 or DMSO vehicle (data are expressed as mean ± SEM; DMSO, *N* = 3; Ceranib-2, *N* = 5 for LAMP-1 and 4 for TFEB; ***P < 0.001 and *P < 0.05 compared to DMSO vehicle control). **(B)** Hematoxylin and Eosin (H&E) staining (upper panel) and immunohistochemistry for CD34 (middle panel) and CD31 (lower panel) in placental sections from Ceranib-2 and DMSO-treated mice. S, spongiotrophoblast layer. L, labyrinthine layer. **(C)** Representative IF images for LAMP-1 (green) and TFEB (green) in placentae of mice injected with either ceranib-2 or DMSO. Nuclei were visualized with DAPI (blue). (–) Non-immune IgG negative control.

We next investigated whether heightened ceramide levels, typical of PE (Melland-Smith et al., 2015), affected lysosomal biogenesis. Treatment of primary isolated trophoblast cells with CER 16:0 significantly increased the total number of lysosomes compared to vehicle treated cells (Figure 3A). To further examine the effect of ceramide on lysosomal biogenesis, we employed trophoblastic JEG3 cells. Pilot titration experiments showed that exposure to 20 μM of CER 16:0 was enough to elicit robust LAMP-1 expression in JEG3 cells (Supplementary Figure 1D). Exposure of JEG3 cells to CER 16:0 and 2-OE (an acid ceramidase inhibitor that increases ceramide levels (Melland-Smith et al., 2015) significantly increased lysotracker signal (Figure 3B). This was accompanied by an increase in TFEB and LAMP-1 proteins (Figure 3C). Furthermore, ceramide treatment induced nuclear localization of TFEB in JEG3 (Figure 3D) and HeLa (Supplementary Figure 2B) cells. Additionally, flow cytometry for Lysotracker^®^ Red and acridine orange corroborated the TEM and lysotracker IF findings. CER 16:0 treatment of JEG3 cells resulted in a much larger population (48.6 vs 6%) of Lysotracker^®^ Red positive cells compared to EtOH vehicle treated cells (Figure 3E, top panels). This was associated with a marked increase in lysosomal volume (Figure 3E, bottom panel). Lysosomal staining with acridine orange corroborated the increased lysosomal volume after CER 16:0 treatment (Supplementary Figure 2A). Negligible shifts in the cell population for both Lysotracker^®^ Red and acridine orange signals were found for Bafilomycin A1-treated JEG3 cells (negative control) and unstained JEG3 cells following CER 16:0 exposure. To confirm the relevance of ceramide on placental lysosomal biogenesis, we used pregnant mice that were injected with Ceranib-2, an inhibitor of acid ceramidase activity. We have previously reported that Ceranib-2 treated mice have increased placental ceramide levels (Melland-Smith et al., 2015), underscoring the utility of this model for examining the impact of elevated ceramide on placental lysosomal biogenesis *in vivo*. WB analysis revealed significant increases in LAMP-1 and TFEB expression in placentae from E13.5 mice injected with Ceranib-2 versus vehicle DMSO (Figure 4A). Haematoxylin and eosin staining showed alterations in the labyrinth layer of the Ceranib-2 injected mice as evident from compaction and decreased vascular branching (Figure 4B-upper panels). This was corroborated by immunohistochemistry for vascular endothelial markers CD34 and CD31 (Figure 4B-middle and bottom panels). In line with human E-PE findings of increased expression in the syncytium (Supplementary Figure 1B,C), LAMP-1 and TFEB levels were elevated in the corresponding labyrinth layer of Ceranib-2 versus vehicle DMSO injected mice (Figure 4C).

**FIGURE 5:**
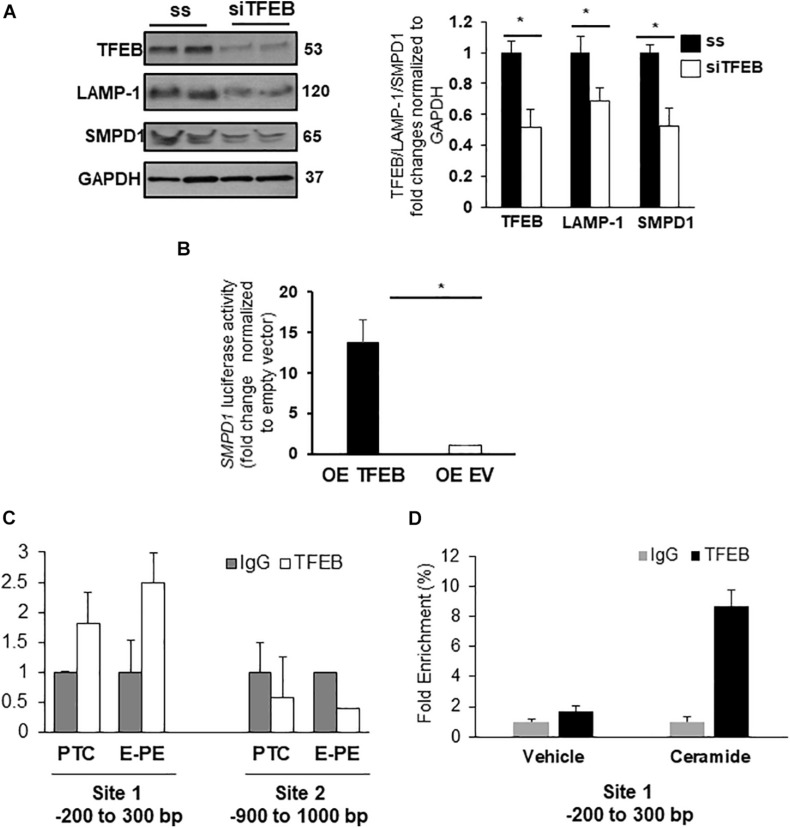
SMPD1 is a direct target gene for TFEB. **(A)** Western blots and densitometric analysis of TFEB, LAMP-1 and SMPD1 in JEG3 cells following transient transfection with *TFEB* siRNA or a control (ss) scrambled sequence (*N* = 3 separate experiments run in duplicate; *P < 0.05 compared to ss control). **(B)** Luciferase reporter assay showing *SMPD1* expression in JEG3 cells following overexpression of *TFEB* (OE TFEB) or empty vector (OE EV). (*N* = 3 separate experiments; *P < 0.05 compared to empty vector control). **(C)** qPCR of *SMPD1* promoter regions –200 to 300 bp and –900 to 1,000 bp after chromatin immunoprecipitation with TFEB in E-PE and PTC placentae (*N* = 4 for each group). **(D)** qPCR of SMPD1 promoter region –200 to 300 bp after chromatin immunoprecipitation with TFEB in JEG3 cells exposed to 20 μM CER16:0 or EtOH vehicle.

Evidence suggests that specific lysosomal hydrolases are under transcriptional regulation of TFEB (Palmieri et al., 2011); however, sphingomyelin phosphodiesterase 1 (SMPD1), a lysosomal enzyme that breaks down sphingomyelin into ceramide (Schuchman and Wasserstein, 2015), has not been examined. *In silico* analysis revealed two putative TFEB binding sites in the *SMPD1* promoter, indicative of its potential regulation by TFEB. RNAi knockdown of *TFEB* in JEG3 cells decreased TFEB, LAMP-1 (established target of TFEB) (Palmieri et al., 2011) and SMPD1 protein levels compared to cells treated with scrambled control RNA (Figure 5A). Using a SMPD1 promoter-driven luciferase reporter construct, we found that concurrent overexpression of TFEB with the SMPD1-luciferase reporter distinctly increased luciferase activity compared to EV control (Figure 5B). To establish TFEB binding to *SMPD1* in PTC and PE placentae, we performed ChIP for TFEB and subsequently examined TFEB-bound *SMPD1* DNA by qPCR. Our data show that TFEB associates with the *SMPD1* promoter at predicted binding site 1 (between −200 to −300 bp) but not at the second binding site (between −900 to −1000 bp) in both PTC and E-PE placentae (Figure 5C). TFEB binding to *SMPD1* promoter was greater in E-PE vs PTC placentae (Figure 5C). Lastly, ChIP analysis confirmed increased TFEB binding to *SMPD1* promoter in JEG3 cells exposed to CER 16:0 (Figure 5D).

### Ceramide Augments TFEB-Induced Lysosomal Exocytosis

**FIGURE 6:**
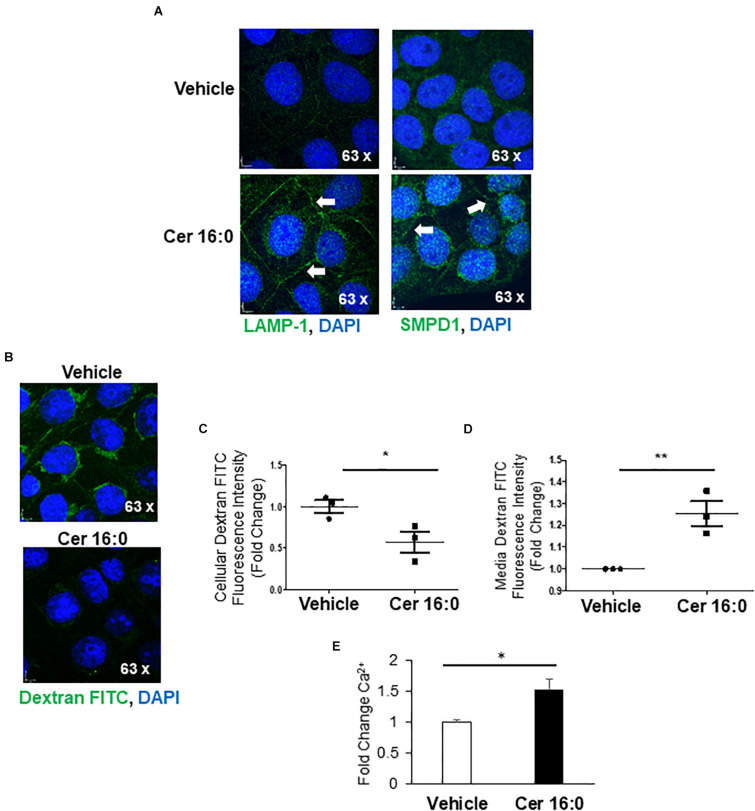
Ceramide triggers lysosomal exocytosis in JEG3 cells. **(A)** IF images for LAMP-1 and SMPD1 in JEG3 cells following exposure to 20 μM CER 16:0 or EtOH vehicle. **(B)** IF images of FITC-dextran (green) loaded JEG3 cells following exposure to 20 μM CER 16:0 or EtOH vehicle. Nuclei: DAPI (blue). **(C)** Fold change in fluorescence intensity of FITC-dextran in JEG3 cells treated with 20 μM CER 16:0 or EtOH vehicle (*N* = 3 separate experiments; *P < 0.05 compared to vehicle). **(D)** Fold change in fluorescence intensity of released FITC-dextran in media of JEG3 cells treated with 20 μM CER 16:0 or EtOH vehicle (*N* = 3 separate experiments; **P < 0.01 compared to vehicle). **(E)** Fold change variation of intracellular Ca^2+^ content of JEG3 cells treated with 20 μM CER 16:0 versus EtOH vehicle. Data are expressed as mean ± SEM.

Immunofluorescence for LAMP-1 and SMPD1 following ceramide treatment revealed increased signals for both proteins in the perinuclear lysosomal compartment and a marked redistribution to the membrane boundaries of the cells treated with ceramide (Figure 6A), indicative of an increased shuttling of lysosomes to the plasma membrane. To investigate the contribution of ceramide to lysosomal exocytosis, we loaded JEG3 cells with FITC-Dextran and measured the release of this saccharide upon ceramide exposure. IF demonstrated a significant decrease of fluorescence in ceramide versus vehicle-treated cells (Figures 6B,C). Furthermore, luminometry revealed a marked increase of fluorescence in the media of ceramide- versus vehicle-treated cells (Figure 6D). Moreover, ceramide increased the intracellular Ca^2+^ content in JEG3 cells (Figure 6E), a potential trigger for exocytosis (Chakrabarti et al., 2003). Together, these observations support the idea that ceramide induces lysosomal exocytosis.

### Accumulation of Ceramide and SMPD1 in Syncytial Apical Villous Membranes

**FIGURE 7:**
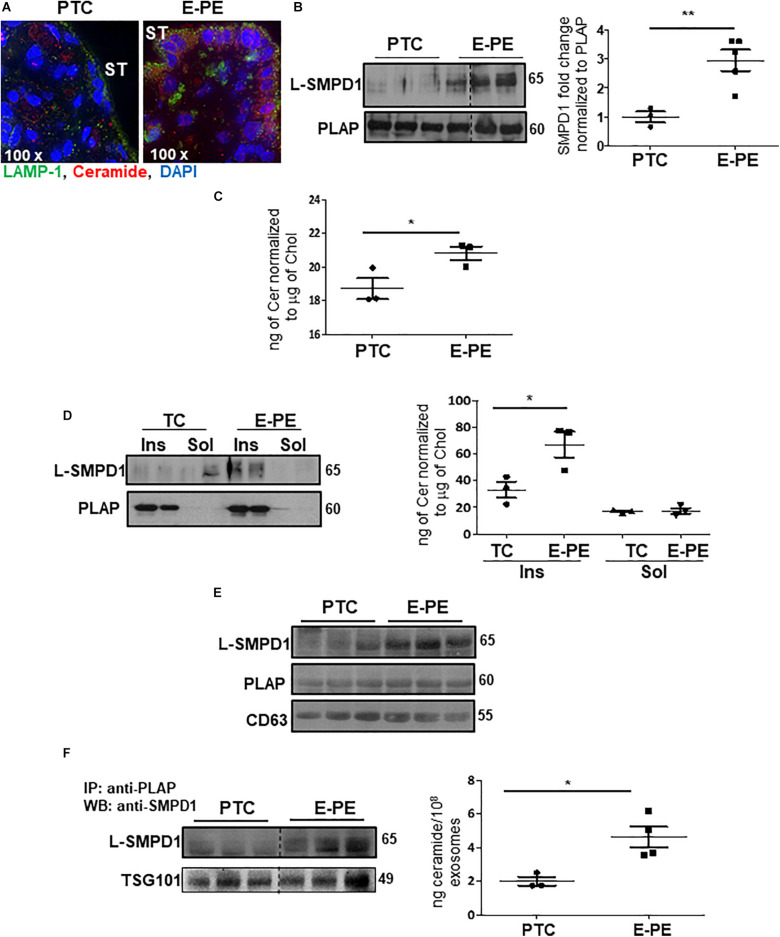
L-SMPD1 localizes to lipid rafts of apical syncytial membranes in E-PE placentae and is released into the maternal circulation *via* exosomes. **(A)** IF images depicting LAMP-1 and ceramide localization in E-PE and PTC placentae. LAMP-1 (green); Ceramide (red); nuclear DAPI (blue). ST, syncytiotrophoblast. **(B)** WB and associated densitometry for L-SMPD1 in lysates of apical syncytial membranes (AM) of PTC and E-PE placentae (PTC, *N* = 3; PE, *N* = 5; **P < 0.01 vs. PTC). PLAP was used as AM marker. **(C)** Total ceramide levels, measured by LC-MS/MS, in syncytial membranes extracts of TC and E-PE placentae (*N* = 3 placentae per group; *P < 0.05 compared to control). (**D-left panel**) Distribution of L-SMPD1 in detergent insoluble (Ins) and soluble (Sol) fractions of AM from TC and E-PE placentae. PLAP was used as lipid raft (detergent insoluble fraction) marker. (**D-right panel**) Total ceramide levels measured by LC-MS/MS in extracts of AM Ins and Sol fractions of TC and E-PE placentae (*N* = 3 samples per group; *P < 0.05 compared to TC). **(E)** WB for L-SMPD1, CD63 and PLAP of exosomes isolated from PTC (*N* = 3) and E-PE (*N* = 3) maternal plasma. **(F)** WB for L-SMPD1 in PLAP-precipitated exosomes from PTC (*N* = 3) and E-PE (*N* = 3) maternal plasma. (**F-right panel**) LC-MS/MS quantification of total ceramide in PTC and E-PE PLAP-precipitated exosomes (E-PE, *N* = 4; PTC, *N* = 3; *P < 0.05 vs. to PTC). Data are expressed as mean ± SEM. Dotted line: non-contiguous lanes run on the same gel.

Immunofluorescence revealed an enrichment of ceramide and LAMP-1 in the syncytiotrophoblast layer of E-PE placentae (Figure 7A). To investigate the occurrence of lysosomal exocytosis in the syncytium, PTC and E-PE placentae were subjected to subcellular fractionation to isolate syncytial apical microvillous membranes (AM), which then were analyzed for the presence of SMPD1. This enzyme is present as a 65 kDa lysosomal form (L-SMPD1) and a 75–80 kDa secreted extracellular form (S-SMPD1) (Jenkins et al., 2010). WB analysis showed the existence of L-SMPD1 in the syncytial AM of E-PE placentae that was significantly increased relative to the syncytial AM of PTC placentae (Figure 7B). To verify that L-SMPD1 was active, we quantified the ceramide content in syncytial AM isolated from E-PE and PTC placentae using tandem mass spectrometry. A significant build-up in ceramide was observed in the syncytial AM of E-PE placentae (Figure 7C). Specifically, we found significant increases in CER 16:0, CER 18:0, and CER 20:0 (Supplementary Figure 3A). Lipid microdomains, termed lipid rafts, are typically enriched in sphingomyelin (Simons and Sampaio, 2011). Hence, we isolated the lipid rafts (detergent-insoluble lipid domains) from the syncytial AM and analyzed them for L-SMPD1 and ceramide content. L-SMPD1 was primarily detected in the placental alkaline phosphatase [PLAP (Ermini et al., 2017)]-positive lipid rafts (insoluble fractions; Ins.) of syncytial AM from E-PE placentae (Figure 7D-left panel). The presence of L-SMPD1 in the lipid rafts was accompanied by heightened ceramide content (Figure 7D-right panel). No differences were found for ceramide content in lipid rafts of TC and PTC AM, suggesting that ceramide enrichment of lipid rafts is independent of gestation (Supplementary Figure 3B,C).

### Ceramide Triggers the Release of L-SMPD1-Enriched Exosomes

**FIGURE 8:**
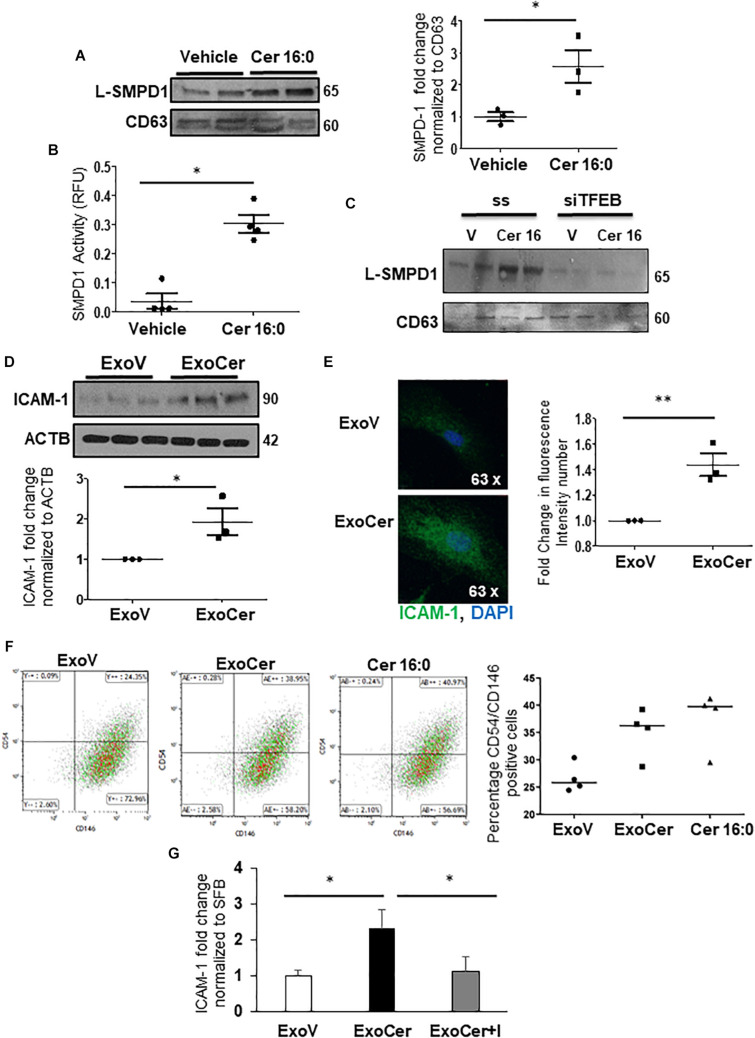
SMPD1 enriched exosomes induces endothelial cells activation. **(A)** WB for L-SMPD1, CD63 and corresponding densitometry of exosomes isolated from conditioned media of JEG3 cells treated with 20 mM CER 16:0 or EtOH (*N* = 3 separate experiments). **(B)** SMPD1 enzyme activity of exosomes from conditioned media of JEG3 cells treated with CER 16:0 or EtOH (*N* = 4 separate experiments). RFU, relative fluorescence units. **(C)** Representative WB for L-SMPD1 of exosomes from JEG3 cells treated with CER 16:0 or EtOH vehicle in conjunction with *TFEB* siRNA or control (ss) scrambled sequence treatment (*N* = 3 separate experiments). **(D)** WB and densitometry of ICAM-1 in lysates of HMVEC cells treated for 3 h with ExoCer or ExoV (*N* = 3 separate experiments). **(E)** IF images and mean fluorescence intensity quantification of ICAM-1 (Green) in HMVEC cells treated with ExoCer or ExoV (*N* = 3 separate experiments). Nuclei: DAPI (blue). (**F-left panels)** Representative flow cytometry density plots of CD54 (ICAM-1) and CD146 of HMVEC cells exposed for 3 h to either 2 × 10^6^ ExoV or ExoCer, or 20 mM CER 16:0. (**F-right panel**) Quantification of CD54^+^/CD146^+^ HMVEC cells after treatments (*N* = 4 experiments). **(G)** Densitometry of WB for ICAM-1 normalized to stain free gel in HMVEC lysates treated for 3 h with ExoV or ExoCer in presence or absence of 25 μM Imipramine (I) (*N* = 3 separate experiments).

We next investigated whether placental L-SMPD1 in E-PE is released into the maternal circulation encapsulated in small nano-sized vesicles. Total and placental exosomes were isolated from maternal plasma of E-PE and normotensive PTC pregnancies (Ermini et al., 2017). WB analysis of L-SMPD1 in total (Figure 7E) and placental (Figure 7F-left panel) exosomes of maternal plasma from E-PE and PTC pregnancies demonstrated a marked enrichment of L-SMPD1 in circulating placental exosomes from E-PE pregnancies. Lipid mass spectrometry showed an increase in ceramides in circulating placental exosomes of PE compared to PTC women (Figure 7F-right panel). Purity of exosome isolation was confirmed by immunoblotting for CD63 and TSG101 (Figures 7E,F). Since ceramide was increased in PE syncytiotrophoblasts, we next investigated if heightened ceramide triggers the exosomal release of L-SMPD1. Exosomes were isolated from media conditioned by JEG3 cells following treatment with CER16:0 or vehicle EtOH. Nanoparticle tracking analysis revealed a significant increase in the number of exosomes (average size of 120 nm) in the conditioned media of JEG3 cells treated with CER16:0 (Supplementary Figure 4A). Consistent with our circulating exosome data from early-onset preeclamptic women (Figures 7E,F), WB showed a significant increase in L-SMPD1 in exosomes isolated from JEG3 cells after ceramide treatment (Figure 8A). The activity of L-SMPD1, indicative of its function, was also significantly greater in exosomes derived from JEG3 cells exposed to ceramide versus vehicle control (Figure 8B). RNAi knockdown of *TFEB* in JEG3 cells markedly decreased L-SMPD1 content in exosomes from cells treated with CER16:0 (Figure 8C), suggesting that the effect of CER 16:0 on exosome L-SMPD1 content is primarily due to TFEB upregulation and activation.

### Exosomal L-SMPD1 Prompts Endothelial Activation and Impairs Angiogenesis

**FIGURE 9:**
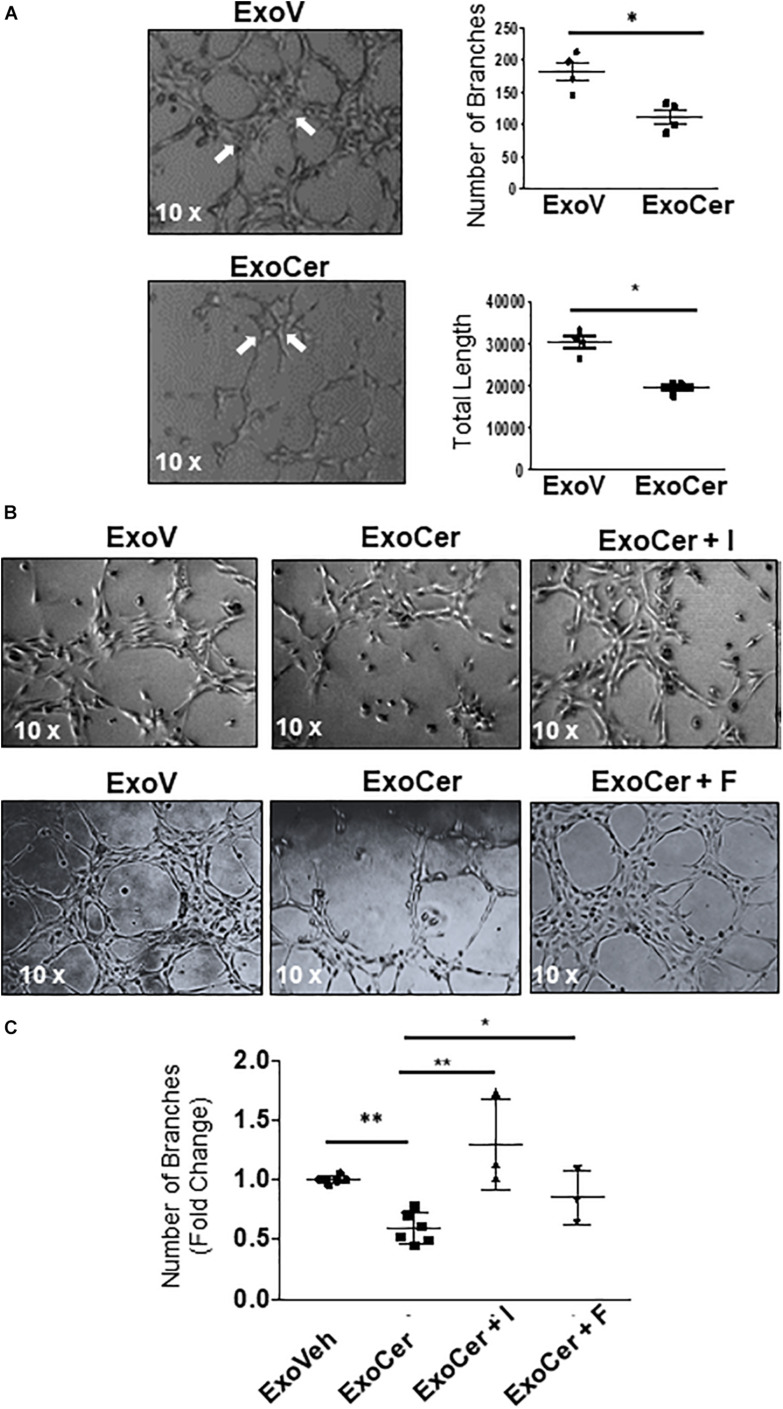
Active SMPD1 in exosomes from JEG3 cells exposed to ceramide affect endothelial angiogenesis. **(A)** Angiogenesis assay of HMVEC treated with 2 × 10^6^ ExoV or ExoCer (*N* = 4 separate experiments). Tube formation was documented after 3 h of treatment by quantification of number of branches and the total length of the segments of the network. Arrows indicates the main branches. Data are expressed as mean ± SEM (*N* = 4 separate experiments). **(B)** Tube formation assay of HMVEC cells treated for 3 h with ExoV or ExoCer in presence or absence of 25 μM Imipramine (I) or 10 μM Fluoxetine (F) and accompanying quantification **(C)** of tubular branches. Data are expressed as mean ± SEM (*N* ≥ 3 separate experiments).

The role of exosomal L-SMPD1 in the development of endothelial dysfunction in preeclamptic women is unknown. Hence, we investigated the effect of SMPD1-enriched exosomes on human microvascular endothelial cells (HMVECs). In pilot experiments, using PKH67 dye-labeled exosomes, we found that exosomal uptake was rapid and peaked between 30–60 min after start of incubation (Supplementary Figure 4B). We then examined whether exosomes derived from JEG3 cells treated with CER 16:0 would affect endothelial activation. HMVECs were treated for 3 h with 2 × 10^6^ exosomes isolated from ceramide (ExoCer) or vehicle (ExoV) treated JEG3 cells. Exposed cells were analyzed for expression of Intercellular Adhesion Molecule 1 (ICAM-1), a marker of endothelial activation (Hunt and Jurd, 1998). Immunoblotting showed a significant increase of ICAM-1 in HMVECs treated with ExoCer compared to those treated with ExoV (Figure 8D). IF for ICAM-1 and relative fluorescence intensity quantification substantiated increased endothelial activation after treatment with ExoCer (Figure 8E). Additionally, flow cytometry for CD54 (I-CAM) and CD146 corroborated the WB and IF findings (Figure 8F). We next examined if treatment of HMVECs with ExoCer disrupted their ability to form tubular endothelial networks in Matrigel. Exposure of HMVECs to ExoCer resulted in a marked reduction of both number of branches and total length of tubules in the endothelial network after 3 h of treatment compared to cells treated with ExoV (Figure 9A). A potential role of L-SMPD1 present in ExoCer on the activation and angiogenesis of HMVECs was investigated using imipramine and fluoxetine, inhibitors of SMPD1 activity (Justice et al., 2018). ExoCer-induced endothelial activation (ICAM-1 expression) was efficiently inhibited by imipramine treatment (Figure 8G). The inhibitory effect of ExoCer on angiogenesis was also attenuated in HMVECs treated simultaneously with either imipramine or fluoxetine (Figures 9B,C). Inhibitors alone had no effect on tube formation of HMVECs (Supplementary Figure 4C). Together, these observations suggest that L-SMPD1 in ExoCer contributes to endothelial dysfunction most likely *via* ceramide generation. In support of this concept, exposure of HMVECs to CER 16:0 and 2-OE (increases intracellular ceramide) resulted in decreased tube formation (Supplementary Figure 4D).

## Discussion

Herein, we demonstrate exuberant lysosomal biogenesis in the syncytium of early-onset preeclamptic placentae that is due to the elevated ceramide build-up in this layer (Melland-Smith et al., 2015). We show that lysosomal SMPD1, a key enzyme for ceramide synthesis, is directly regulated by TFEB. In addition, we report that lysosomal exocytosis is prominent in PE. It enriches the apical membranes of the syncytial layer with lysosomal SMPD1. The presence of this enzyme in the lipid rafts of apical syncytial membranes from E-PE placentae increases the ceramide content in these membrane domains leading to the release of ceramide-enriched placental exosomes containing active lysosomal SMPD1 into the maternal circulation. L-SMPD1 in the released exosomes promotes endothelial activation and impairs angiogenesis, thereby contributing to the endothelial dysfunction in E-PE women.

In the present study, we show increased number of lysosomes in trophoblast cells of E-PE placentae mostly due to a striking increase in secondary lysosomes. This pool of lysosomes is central to the degradation and recycling of macromolecules delivered by endocytosis and autophagy. This increase in lysosomes in E-PE agrees with numerous reports of elevated autophagy in preeclamptic placentae (Oh et al., 2008; Kalkat et al., 2013; Akaishi et al., 2014; Gao et al., 2015; Melland-Smith et al., 2015; Akcora Yildiz et al., 2017; Hutabarat et al., 2017; Ausman et al., 2018; Zhao et al., 2020). Lysosome biogenesis is regulated by a lysosome-to-nucleus signaling mechanism that involves TFEB. Under stress conditions, TFEB translocates to the nucleus, where it promotes its own expression as well as that of genes involved in lysosomal biogenesis and autophagy (Roczniak-Ferguson et al., 2012; Martina and Puertollano, 2017). Here, using siRNA knockdown, reporter gene assay and ChIP analysis, we show that the *SMPD1* gene is a direct target of TFEB. In support of TFEB stimulating autophagy in E-PE placentae, we demonstrate increased expression of autophagy genes BECN1 and ATG9b. Both are established TFEB targets (Palmieri et al., 2011; Zhang et al., 2019). Mice lacking TFEB die *in utero* between E9.5 and E10.5 days and exhibit severe defects in placental vascularization, underscoring the significance of this transcription factor for placental development (Steingrimsson et al., 1998). In the present study, we show that TFEB and LAMP1 expression is increased in human E-PE placentae compared to TC and PTC placentae, in line with the observed increase in number of lysosomes. In contrast, Nakashima et al. (2020) reported that autophagolysosomal degradation is defective in preeclamptic placentae due to reduced TFEB and LAMP1 expression. Although their finding may explain the build up of protein aggregates in preeclamptic placentae, it clashes with the generally accepted view of elevated autophagy flux in preeclamptic placentae (Oh et al., 2008; Kalkat et al., 2013; Akaishi et al., 2014; Gao et al., 2015; Melland-Smith et al., 2015; Akcora Yildiz et al., 2017; Hutabarat et al., 2017; Ausman et al., 2018; Zhao et al., 2020). Unfortunately, the authors did not verify their findings at the ultrastructural level (TEM is the gold standard for surveillance of organelles and autophagy) and mainly focused on extravillous trophoblasts while we surveyed the villous trophoblast layers. Moreover, they employed a limited number of cases for both late-onset and, particularly, early-onset preeclamptic placentae, whereas we used a large number (*n* = 54) of strictly early-onset preeclamptic placentae.

We have reported lysosomal accumulation of ceramide in PE syncytium (Melland-Smith et al., 2015). Studies in retinal epithelial cells have shown that ceramide induces TFEB translocation to the nucleus (Martina and Puertollano, 2018). Treatment of pre-osteoblastic cells with the ceramide analog PDMP, indirectly activated TFEB, thereby triggering autophagy (Ode et al., 2017). Our findings of heightened levels of TFEB in ceramide-treated JEG3 cells and in placentae of Ceranib-2 injected mice, agree with these observations. Ensuing increased lysosome biogenesis was corroborated by markedly augmented lysosome number, lysosomal volume and lysosomal marker expression in primary isolated trophoblast cells and JEG3 cells following ceramide and 2-OE treatments.

Transcription factor EB functions as a key regulator of lysosomal exocytosis by increasing the pool of lysosomes in proximity of the plasma membrane (Medina et al., 2011). In the present study, we demonstrated that ceramide, besides increasing nuclear TFEB, also stimulated the redistribution of lysosomal proteins (LAMP-1 and L-SMPD1) to the membrane boundary of JEG3 cells. An increase of LAMP-1 in the plasma membrane has also been reported for fibroblasts following sucrose challenge (Samarani et al., 2018). Our findings confirm that lysosomal exocytosis in JEG3 cells is activated upon ceramide treatment. Increased exocytosis is supported by an increase in intracellular calcium (Chakrabarti et al., 2003). In PE placentae, we found lysosomal SMPD1 in the syncytial AM, suggesting active lysosomal exocytosis in this layer *in situ*. Hence, it is plausible that high ceramide content in E-PE lysosomes triggers their biogenesis to clear ceramide *via* exocytosis.

Sphingomyelins are uniquely enriched in membrane subdomains named lipid rafts (Simons and Sampaio, 2011). Our finding of lysosomal SMPD1 in lipid rafts of syncytial AM from E-PE placentae suggest that this enzyme is likely responsible for the increase of ceramides in these microdomains. Accumulation of ceramide within syncytial lipid rafts plausibly contributes to the heightened autophagy of the syncytium, a characteristic of E-PE pathology (Kalkat et al., 2013; Melland-Smith et al., 2015; Ausman et al., 2018). Ceramide build-up in lipid rafts has been reported to induce internal curvature of the membranes thereby facilitating endocytosis and consequently fusion with lysosomes leading to increased rates of autophagy (Cremesti et al., 2002; Tam et al., 2010). The majority of studies have linked heightened autophagy in trophoblast layers (i.e., syncytium) of preeclamptic placentae to accelerated cell death (Oh et al., 2008; Kalkat et al., 2013; Akaishi et al., 2014; Gao et al., 2015; Melland-Smith et al., 2015). However, one opposing report suggests that autophagy is needed for extravillous trophoblast invasion and migration into the myometrium (Nakashima et al., 2013, 2017). Thus, it is possible that lysosome biogenesis and exocytosis may differ in different subpopulations of trophoblast cells.

Recent proteomic studies have found SMPD1 in exosomes isolated from prostatic secretions in urine (Wang et al., 2012; Principe et al., 2013). In the present study, we provide evidence that active L-SMPD1 is present in placental exosomes isolated from maternal plasma of E-PE women and from JEG3 cells following ceramide exposure. It is likely that L-SMPD1 in lipid rafts of the syncytial plasma membrane of PE placentae degrades sphingomyelin to ceramide, thereby enriching these membrane microdomains with ceramide. These ceramide-enriched lipid rafts can easily bud from the inner plasma membrane to form early endosomes that when cholesterol-rich are routed *via* the multivesicular bodies (MVB) pathway (Raposo and Stoorvogel, 2013) for secretion of exosomes containing active L-SMPD1.

It is well known that extracellular vesicles are involved in cell-to-cell communication by delivering proteins, lipids and RNA that can alter the physiological status of recipient cells (Maas et al., 2017). *In vitro* studies have indicated that microvesicles circulating in maternal blood of women with PE affect angiogenesis and function of HUVECs (Shomer et al., 2013; Escudero et al., 2016). However, the exact cargo affecting endothelial function remains to be established. Herein, we show that L-SMPD1 is present and active in circulating placenta-derived exosomes of preeclamptic women. Additionally, we show that lysosomal SMPD1-containg exosomes alter endothelial function *in vitro*. Interestingly, secretory SMPD1 activity has been reported to be elevated in first trimester plasma of women that later develop late-onset PE (Rodriguez-Sureda et al., 2016).

Various reports suggest that ceramide impairs vascular reactivity (Alewijnse and Peters, 2008; Spijkers et al., 2010). Endogenous formation of ceramide due to increased SMPD1 activity has been shown to trigger pro-inflammatory responses (Modur et al., 1996). Evidence also indicates that SMPD1 activity is required to facilitate T cell adhesion mediated by ICAM-1 and consequently transmigration across brain endothelial cells (Lopes Pinheiro et al., 2016). In the present study, we found a significant increase of ICAM-1 in HMVECs treated with SMPD1-enriched exosomes derived from ceramide-treated trophoblastic JEG3 cells. The exosome-induced endothelial (ICAM-1) activation was blocked by the SMPD1 inhibitor Imipramine, suggesting a role for exosomal L-SMPD1 in this process. Exosomes isolated from the maternal circulation of preeclamptic women have been shown to reduce angiogenesis of HUVEC cells (Chang et al., 2018) but the exosomal cargo causing this effect remains unknown. Treatment of HUVEC cells with synthetic CER 2:0 and 6:0 have been reported to alter their tubule formation ability (Bansode et al., 2011; Mehra et al., 2014). Our present finding of limited tubule formation of HMVECs in the presence of CER 16:0 and acid ceramidase inhibitor 2-OE agree with the latter observation. Lysosomal SMPD1-containing exosomes derived from ceramide-treated trophoblast cells also reduced the angiogenesis of HMVEC, an effect that was abrogated by SPMPD1 inhibitors Imipramine and Fluoxetine. Based on these observations, we postulate that ceramide provokes L-SMPD1 accumulation as cargo in circulating placental exosomes of preeclamptic women thereby triggering maternal endothelial dysfunction. A hallmark of endothelial dysfunction is reduced nitric oxide (NO) bioavailability. Ceramide has been shown to reduce NO synthesis and bioavailability (Li et al., 2002; Symons and Abel, 2013). Thus, it is plausible that fusion of circulating placental exosomes containing active L-SMPD1 with maternal vascular cells in PE women leads to aberrant ceramide generation that reduces NO availability, culminating in endothelial dysfunction. Considering the importance of ceramide for exosomal formation, secretion and signaling, future studies aimed at targeting ceramide homeostasis may prove valuable to ameliorate maternal systemic endothelial dysfunction characterizing PE.

In summary, we postulate that oxidative stress conditions, typical of early-onset PE leads to ceramide build up (Melland-Smith et al., 2015) that, in turn, triggers TFEB expression. Preliminary findings suggest that oxidative stress may directly affect TFEB expression and activation in trophoblast cells (Supplementary Figure 2C) but involved signaling pathways need further investigation. Thus, it is plausible that both oxidative stress and its downstream mediator ceramide contribute to the heightened lysosomal biogenesis and exocytosis found in E-PE. The latter causes lysosomal SMPD1 to transfer to the syncytial plasma membrane resulting in the formation of ceramide-enriched lipid microdomains, which are more prone to membrane budding and exosome biogenesis leading to the release of SMPD1 into the maternal circulation.

## Data Availability Statement

The raw data supporting the conclusions of this article will be made available by the authors, without undue reservation.

## Ethics Statement

The studies involving human participants were reviewed and approved by the Mount Sinai Hospital Research Ethics Board (REB number: 11-0287-E). The patients/participants provided their written informed consent to participate in this study. The animal studies were reviewed and approved by the Animal Care Committee of the Hospital for Sick Children (Toronto, Canada).

## Author Contributions

LE and AF: conceptualization, formal analysis, investigation, methodology, validation, visualization, and writing – original draft preparation. SA: formal analysis, investigation, methodology, and writing – review and editing. JA, CP, and JS: formal analysis, investigation, methodology, and validation. MM-S: investigation and methodology. ME, TP, and ML: methodology and validation. ON: methodology. MP: conceptualization, funding acquisition, methodology, and writing – review and editing. IC: conceptualization, data curation, formal analysis, funding acquisition, methodology, project administration, resources, supervision, and writing – review and editing. All authors contributed to the article and approved the submitted version.

## Conflict of Interest

The authors declare that the research was conducted in the absence of any commercial or financial relationships that could be construed as a potential conflict of interest.
