# Supplementary material for: Ceramide-Induced Lysosomal Biogenesis and Exocytosis in Early-Onset Preeclampsia Promotes Exosomal Release of SMPD1 Causing Endothelial Dysfunction
Source: Front Cell Dev Biol. 2021 May 4;9:652651. doi: 10.3389/fcell.2021.652651 (PMC8130675; doi:10.3389/fcell.2021.652651)
Supplement: Supplementary file 1 [file Data_Sheet_1.PDF]

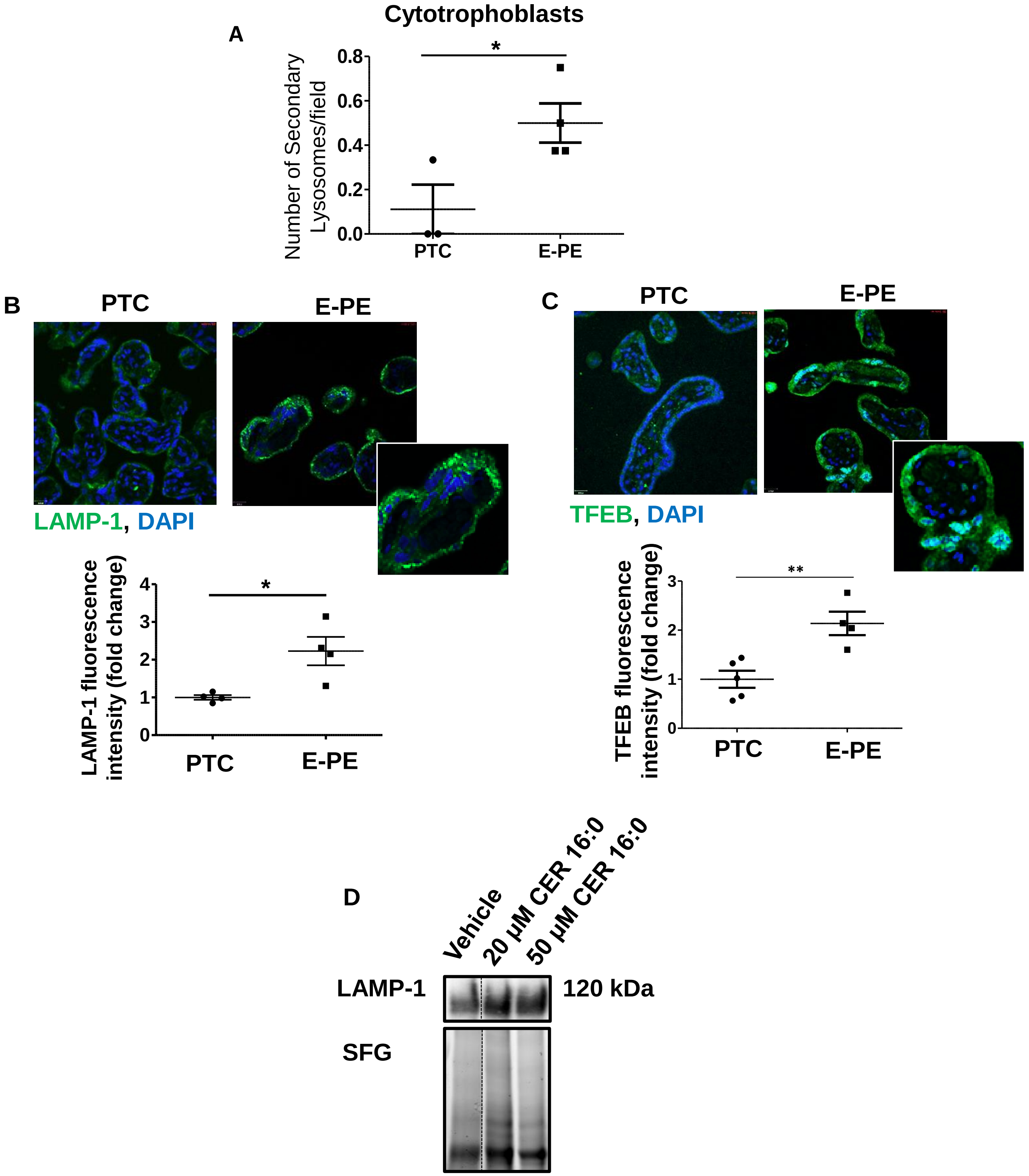

Supplementary Figure 1. (**A**) Secondary (right panel) lysosome number in cytotrophoblast cells from PCT and PE placentae (PE, N =4; PTC, N =3; *P < 0.05 compared to PTC). (**B**) Representative IF images and mean fluorescence intensity quantification of LAMP-1 in PTC and PE placentae (PE, N=4; PTC, N=4; *P < 0.05 compared to PTC). (**C**) Representative IF images and mean fluorescence intensity quantification of TFEB in PTC and PE placentae (PE N=4, PTC N=5; **P < 0.01 compared to PTC). Nuclei were visualized with DAPI (blue). (**D**) Representative WB for LAMP-1 in JEG3 cells treated with 20 and 50 µM of CER 16:0 showing a dose-response. Stain free Gel (SFG) demonstrating total protein. Data are expressed as mean ± SEM.

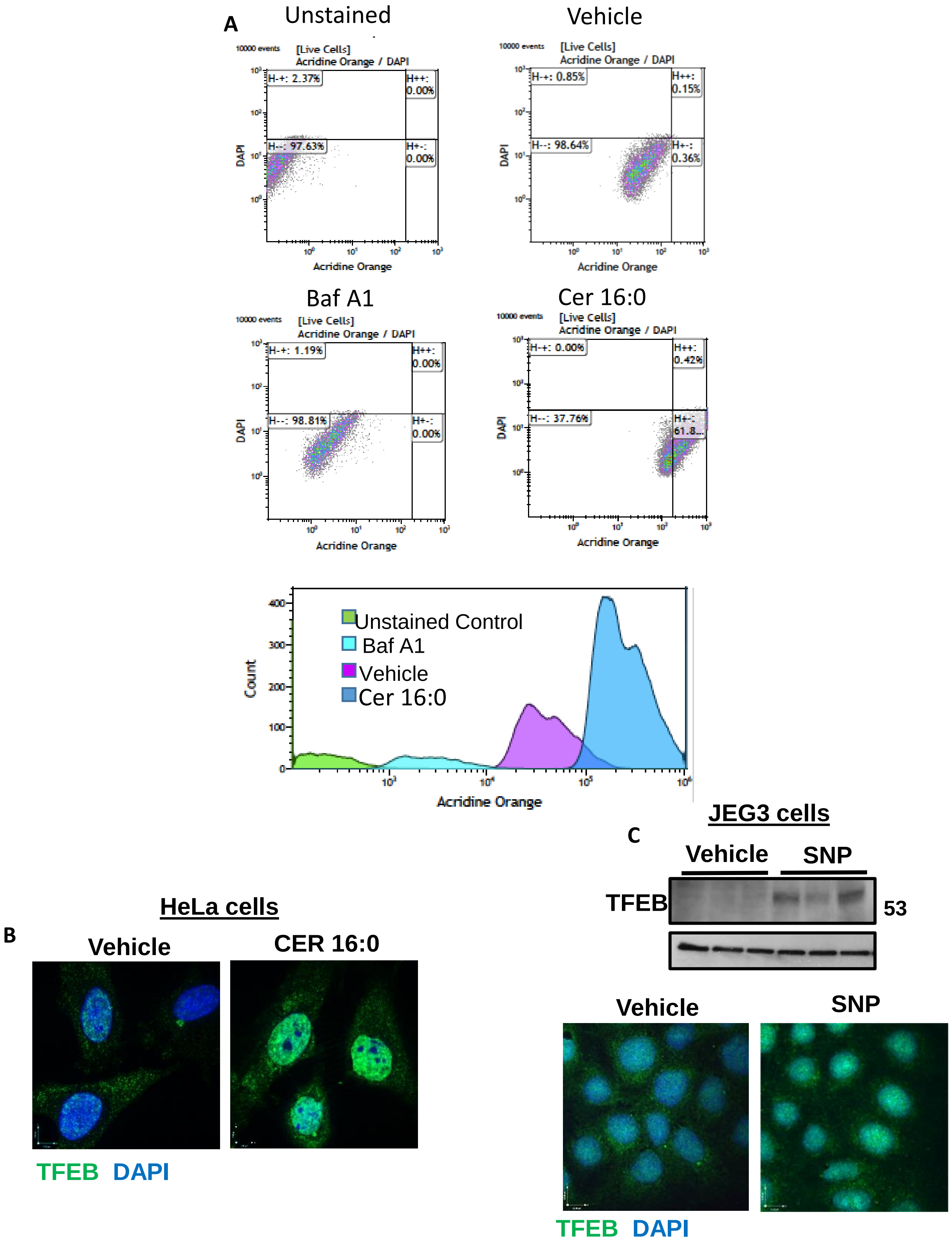

Supplementary Figure 2. (**A**) Representative flow cytometry density plots of acridine orange in JEG3 cells exposed for 6h to either, EtOH vehicle, 100nM Bafilomycin (Baf A1) or 20 mM CER 16:0. Negative control, unstained cells. Note that DAPI staining, used to mark viability of live cells indicate no significant shifts between unstained, vehicle EtOH, CER16:0 or Baf A1. (**B**) Representative IF images for TFEB (green) in HeLa cells treated with 2.5 mM SNP or vehicle for 24 h. DAPI staining identifies nuclei. (**C**)Representative WB and IF for TFEB in JEG3 cells treated with 2.5 mM SNP or vehicle for 24 h. DAPI staining detects nuclei.

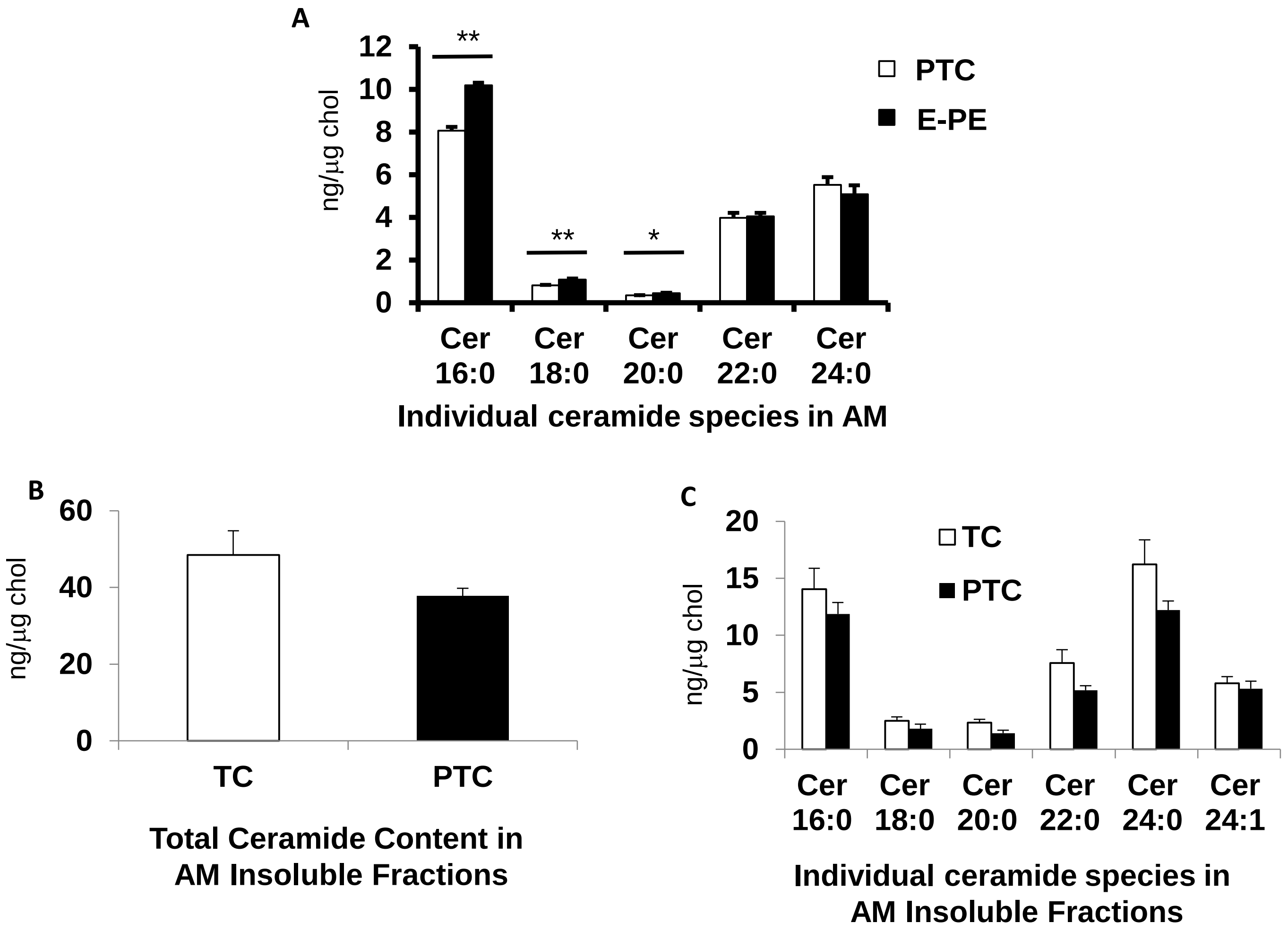

Supplementary Figure 3. (**A**) Quantification of ceramides by LC-MS/MS in apical (syncytial) membrane enriched fractions of TC and PE placentae (N = 3 placentae for group; *P < 0.05, **P< 0.01). (**B** and **C**) Quantification of total ceramide and ceramide species by LC-MS/MS in apical (syncytial) membrane enriched fractions of TC and PTC placentae (N = 4 placentae for group). Data are expressed as mean ± SEM.

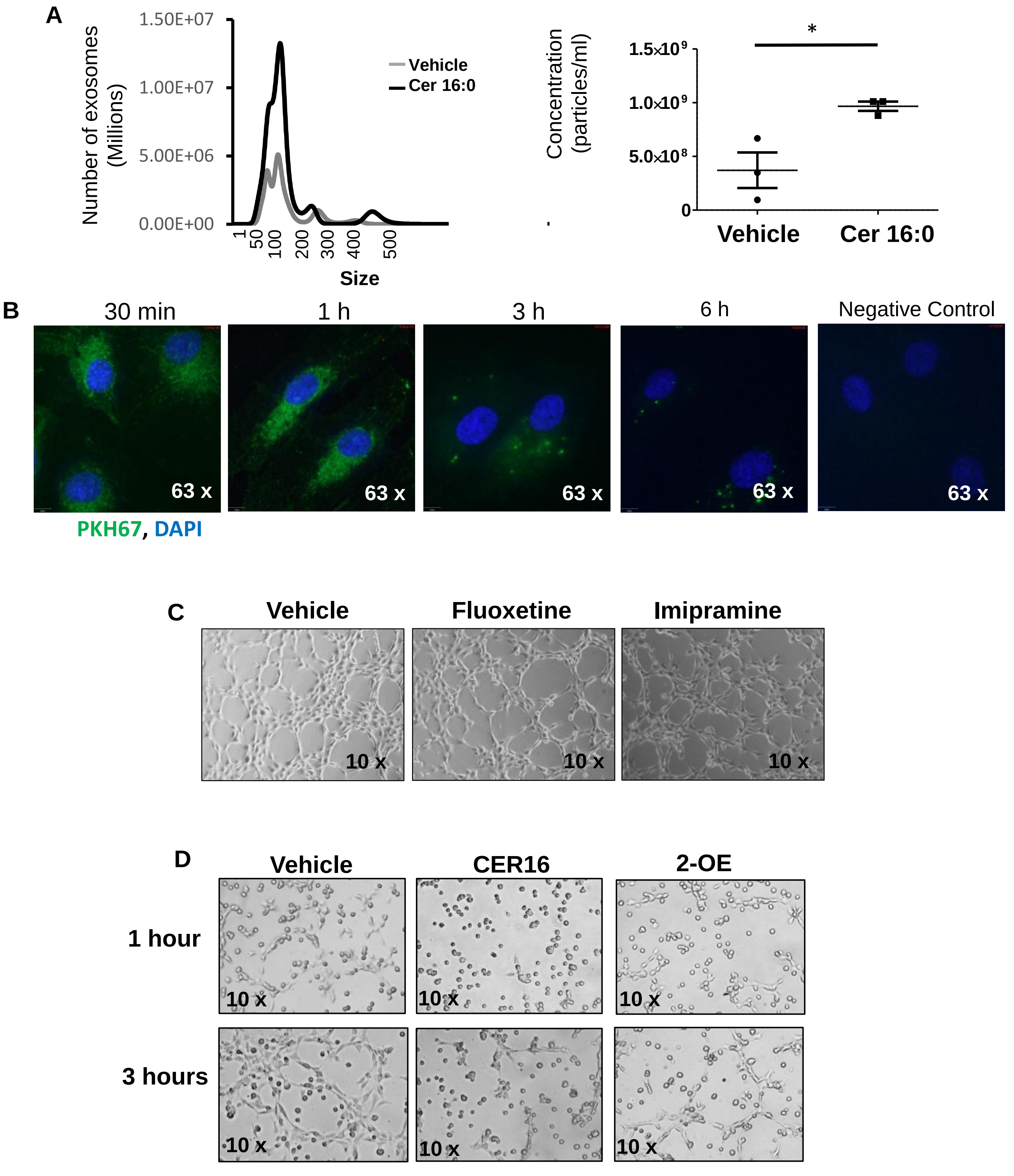

Supplementary Figure 4. (**A**) Size distribution and quantification of exosomes isolated from the conditioned media of JEG3 cells treated with 20 µM CER 16:0 or vehicle ethanol (N = 3 separate experiments; *P < 0.05). Data are expressed as mean ± SEM. (**B**) Exosome uptake by HMVEC, exosomes isolated from JEG3 conditioned media were labelled with PKH67 (green). HMVEC were subsequently incubated with the labelled nanovesicles for different durations. Negative control: unlabelled exosomes. (**C**) Tube formation assay in HMVEC cells exposed to vehicle, 10uM fluoxetine and 25uM imipramine. (**D**) Tube formation assay of HMVEC cells treated with vehicle, 20 µM Ceramide 16:0 or 25 µM 2-OE.
